# Retrieving and processing agro-meteorological data from API-client sources using R software

**DOI:** 10.1186/s13104-021-05622-8

**Published:** 2021-05-26

**Authors:** Adrian A. Correndo, Luiz H. Moro Rosso, Ignacio A. Ciampitti

**Affiliations:** Department of Agronomy, Kansas State University, Manhattan, KS 66506 USA

**Keywords:** Programming, Agriculture, Daymet, Nasapower, Chirps

## Abstract

**Objectives:**

The main purpose of this publication is to help users (students, researchers, farmers, advisors, etc.) of weather data with agronomic purposes (e.g. crop yield forecast) to retrieve and process gridded weather data from different Application Programming Interfaces (API client) sources using R software.

**Data description:**

This publication consists of a code-tutorial developed in R that is part of the data-curation process from numerous research projects carried out by the Ciampitti’s Lab, Department of Agronomy, Kansas State University. We make use of three weather databases for which specific libraries were developed in R language: (i) DAYMET (Thornton et al. in https://daymet.ornl.gov/, 2019; https://github.com/bluegreen-labs/daymetr), (ii) NASA-POWER (Sparks in J Open Source Softw 3:1035, 2018; https://github.com/ropensci/nasapower), and (iii) Climate Hazards Group InfraRed Precipitation with Station Data (CHIRPS) (Funk et al. in Sci Data 2:150066, 2015; https://github.com/ropensci/chirps). The databases offer different weather variables, and vary in terms of spatio-temporal coverage and resolution. The tutorial shows and explain how to retrieve weather data from multiple locations at once using latitude and longitude coordinates. Additionally, it offers the possibility to create relevant variables and summaries that are of agronomic interest such as Shannon Diversity Index (SDI) of precipitation, abundant and well distributed rainfall (AWDR), growing degree days (GDD), crop heat units (CHU), extreme precipitation (EPE) and temperature events (ETE), reference evapotranspiration (ET0), among others.

## Objective

The objective of this dataset [1] containing a code-tutorial is to assist end users to retrieve and process gridded weather data using R software. This information can facilitate the collection of a diverse number of weather data parameters from multiple locations, in addition to assist on the rapid intake of data for multiple farming modeling systems to improve geo-spatial simulations considering weather as a key factor and to help us for scaling the results from multiple research projects. Two pertaining examples of the application of the code can be found in:

Correndo et al. [2]. *Assessing the uncertainty of maize yield with no nitrogen fertilization.* In Correndo et al. [2], gridded weather data from Daymet was obtained for 679 site-years across North America. Variables were summarized into monthly periods during the cropping season on maize, with the purpose of predicting the crop yield using a machine learning algorithm (conditional random forests).

Borja Reis et al. [3]. *Environmental factors associated with nitrogen fixation prediction in soybean*. In Borja Reis et al. [3], the code was used to obtain and process weather data for 95 site-years across the United States. Variables were summarized into custom periods (based on phenological stages) during the soybean cropping season, with the purpose of predicting soybean biological nitrogen fixation using a machine learning model (Elastic net).

## Data description

All data files are deposited in the Harvard Dataverse repository, dataset “Agrometeorological data using R-software” [1]. The programming code (Data file 1 in Table 1) serves to retrieve weather data from multiple API-client sources and to produce secondary variables that are meaningful in agronomic terms. The R-code (*.rmd) was generated using R version 4.0.3 (Linux-GNU, 64-bit) and R-studio v1.2.5042. However, additional machine specifications are not required in order to execute the R code. The time will be dependent on the volume of data (number of locations) and internet connection.

**Table 1:** Overview of data files/data sets

Label	Name of data file/data set	File types (file extension)	Data repository and identifier (DOI or accession number)
Data file 1	code_agromet_R	RMarkdown file (.rmd)	Harvard Dataverse: 10.7910/DVN/J9EUZU [1]
Data file 2	Tutorial_agromet_R	PDF file (.pdf)	Harvard Dataverse: 10.7910/DVN/J9EUZU [1]
Dataset 1	Example_input	CSV file (.csv)	Harvard Dataverse: 10.7910/DVN/J9EUZU [1]
Dataset 2	Example_input_historical	CSV file (.csv)	Harvard Dataverse: 10.7910/DVN/J9EUZU [1]

The API-client sources [4–9] offer different variables by default. Particularly, DAYMET offers the best combination of agrometeorological variables at the highest spatial resolution (~ 1 km^−2^). However, the geographical coverage of DAYMET only includes North America. At a global scale, NASA-POWER offers the most complete set of variables, nonetheless, at a much lower spatial resolution (~ 50 km^−2^). Lastly, CHIRPS offers global data (− 50 to + 50 latitude degrees) constrained to precipitations, however, with a better spatial resolution (~ 5 km^−2^) than NASA-POWER.

In the tutorial file (Data file 2 in Table 1): (i) we provide extended explanations along with the lines of code showing how to download and process daily-weather data (Section 2 of the code), and (ii) we offer the option to generate new variables and summaries for different time intervals during the cropping season or historical periods (Sections 3 to 5 of the code). Details of calculations of secondary variables and summary options are provided in the Tutorial file.

The data table files (Dataset 1 and Dataset 2 in Table 1) represent examples of data inputs the user needs to provide in order to make the request of weather data to the data servers.

## Limitations

- The code may be limited in the number of variables, which may not satisfy specific needs.
- Thermal time variables (growing degree days and Crop Heat Units) presented in the example are just for reference, using specifications for *Zea mays* L. (corn, maize). For other crops, user should manually modify the specific lines of code.
- Although the Parameter elevation Regression on Independent Slopes Model (PRISM) database is available in R-software (https://docs.ropensci.org/prism/), it does not allow users to *directly* retrieve multi-location weather data using latitude and longitude coordinates. For this reason, the PRISM library was not included on this tutorial. Next versions are likely to include the option of using PRISM.

## Data Availability

The data described in this Data note can be freely and openly accessed at Harvard Dataverse: 10.7910/DVN/J9EUZU [1]. Please see Table 1 and references [1] for details and links to the data.

## Abbreviations

CHIRPS: Climate Hazards Group InfraRed Precipitation with Station Data
SDI: Shannon Diversity Index
AWDR: Abundant and well distributed rainfall
GDD: Growing degree days
CHU: Crop heat units
ETE: Extreme precipitation events (EPE)
ETE: Extreme temperature events
ET0: Reference evapotranspiration
